# How big a drop in agricultural exports to the United Kingdom after Brexit? Simulations for sensitive products of four Visegrad countries

**DOI:** 10.1371/journal.pone.0274462

**Published:** 2022-09-20

**Authors:** Karolina Pawlak, Jan Hagemejer, Jan Jakub Michalek, Maria Dunin-Wasowicz

**Affiliations:** 1 Department of Economics and Economic Policy in Agribusiness, Faculty of Economics, Poznan University of Life Sciences, Poznan, Poland; 2 Department of Macroeconomics and International Trade Theory, Faculty of Economic Sciences, University of Warsaw, Warsaw, Poland; 3 CASE Center for Social and Economic Research, Warsaw, Poland; 4 European Movement Forum, Warsaw, Poland; University of Macerata, ITALY

## Abstract

After the European Union (EU) was left by the United Kingdom (UK), a free trade area was established between these economies. Although bilateral trade in all goods is tariff-free, regulatory requirements make exports more costly and burdensome. We used a partial equilibrium model to analyze the implications of Brexit for agricultural exports from Visegrad countries (Czechia, Hungary, Poland and Slovakia). We assess trade creation and trade diversion effects resulting from an increase in non-tariff measures and border costs for 4-digit agricultural products identified as sensitive in each of the Visegrad countries. The simulations reveal that exports of sensitive products from Visegrad countries to the UK could decrease by up to 20%. While the macroeconomic importance of this change is not significant, for the producers of the sensitive goods such export losses are substantial.

## Introduction

Following the referendum on 23 June 2016, the United Kingdom (UK) voted to leave the European Union (EU). Leaving the EU is likely to have significant implications for the agricultural sector and agri-food trade both in the UK and the countries remaining in the EU, including the Visegrad (V-4) countries (Czechia, Hungary, Poland and Slovakia). Membership in the EU affects farming in a number of ways, including the impact of the Common Agricultural Policy (CAP) and trade arrangements [1]. As in all EU trade negotiations, due to the specific nature of agricultural products, the complicated instruments of the CAP and the importance of agriculture to the national economies, agricultural matters became one of the difficult issues in the EU-UK trade negotiations (for more on the specificity of preferential liberalization of trade in agricultural products see e.g. [2–11]). On 24 December 2020, the new Trade and Cooperation Agreement (TCA, or so-called Brexit Agreement–BA) was reached between the UK and the EU, and it was signed by both parties on 30 December 2020. It was provisionally applied from 1 January 2021, and entered into force on 1 May 2021. It is still too early to provide an ex-post evaluation of the trade changes resulting from Brexit, due to the lack of sufficient statistical data and the overlapping effects of the coronavirus pandemic. Indeed, the commencement of EU-UK Withdrawal Agreement (WA) negotiations in June 2017 [12] was conducive to undertaking a number of new analyses employing partial (PE) and general equilibrium (GE) models to evaluate the economic consequences of Brexit. Intensive research related to the economic effects of Brexit had already started in 2012. A brief review of the most important findings in the field is presented by Hagemejer et al. [13].

Analyses of Brexit carried out to date have usually predicted moderate changes in EU trade in agri-food products, ranging from 3 to 11%, depending on the adopted scenario (from a single market to non-preferential WTO rules). A much greater decrease in the value of exports and imports, exceeding 60%, was estimated for the UK by Choi et al. [14]. Bellora et al. [15] estimated that Brexit would affect the largest exporters to the UK, including the Netherlands, Ireland and France. Possible trade impacts of Brexit on the Dutch and Irish agri-food sectors were studied by Donnellan and Hanrahan [16], Rojas-Romagosa [17] and van Berkum et al. [18]. The literature on the subject also includes estimates of the trade effects of Brexit on Denmark [19, 20], while analyses for countries in the Central and Eastern Europe (CEE) region, including the V-4 countries, are lacking. Existing descriptive studies by Vasary [21] and Zawojska [22] pointed out that under a “no-deal” Brexit scenario, the UK market could become much less attractive for Visegrad agri-food exporters, particularly in the case of bovine, pork and dairy products. In addition, a potential reduction in current UK tariff protection against third countries, and an increase in non-tariff measures (NTMs) in trade flows between the EU and UK, would raise the relative costs of Visegrad exporters. In the light of the above, there is a research gap in the field of quantitative assessment of possible trade effects of Brexit on agri-food trade in the V-4 countries.

In this paper we address this gap and analyze the implications of the Brexit Agreement in regard to agricultural exports from V-4 countries. For this purpose, we perform simulations using a partial equilibrium model. Our simulation scenario is based on the outcome of the Brexit negotiations indicated, i.e. we assume the existence of the free trade area (FTA), but with no specific commitments regarding non-tariff measures. Moreover, we assume a substantial increase in border costs, resulting from leaving the Single European Market (SEM) and the introduction of border checks.

We simulate the increase in NTMs resulting from a possible divergence of regulatory standards and the increase in border costs differentiated by agricultural sectors. We use tariff equivalents of NTMs estimated separately using a gravity model and GTAP bilateral trade data.

We identify the 4-digit sensitive agricultural product groups for each V-4 country. These products have a large share (more than 0.5%) in these countries’ exports and face a significant increase in NTM tariff equivalents and border costs. Sensitive products are defined as those most vulnerable to changes in trade policies. According to the WTO [23], for this limited number of products, countries could offer market access improvements through a combination of smaller tariff reductions and tariff quotas setting instead of full tariff reduction under a tiered formula. The pattern of sensitive products differs between the V-4 countries. In the case of Poland, the structure of exports is dispersed and covers 18 sensitive groups, while for the other three countries their exports are much more concentrated within two or three 4-digit groups of the HS trade classification. We perform simulations for 6-digit HS groups and afterwards aggregate results to 4-digit HS groups. We analyze trade creation and diversion effects resulting from the increase of NTMs and border costs.

The paper is organized into seven sections, including this introduction. In section 2 we present the major provisions of the EU-UK Withdrawal Agreement referring to agricultural trade. In section 3 we provide a brief review of literature. Section 4 discusses the value and structure of agri-food trade between V-4 countries and the UK. In section 5 we provide a brief description of the research method, including the simulation scenario and structure of the SMART partial equilibrium model used. Section 6 presents and discusses the results of our simulations. The last section concludes and summarizes the main findings.

## The EU-UK withdrawal agreement

The UK submitted its formal request to exit the EU in March 2017, and several weeks later it initiated the process of negotiations with the EU-27 on the EU-UK Withdrawal Agreement (WA) and on the future economic relationship. The Directives for the negotiation for the Withdrawal Agreement were given by the EU Council of European Union in the document: XT 21016/17 ADD 1 REV 2, dated 22 May 2017. The Political Declaration issued in 2018, setting out the framework for the future bilateral relationship, was very optimistic and described a future agreement on deep integration.

Ultimately both parties signed the WA in November 2019 [24]. The EU and the UK had jointly agreed on a one-year transition period, which lasted until 31 December 2020. The United Kingdom left the European Union on 31 January 2020. Since this date, the UK has officially been a third country to the EU and hence no longer participates in the Single European Market (SEM). At the same time the UK entered negotiations for the so-called Brexit Agreement (BA), as mentioned earlier, with the EU. Due to the political tensions between the EU and UK, as well as within the UK Parliament, the option of a “very soft” BA was excluded. At the very last moment, on 24 December 2020, the EU and the UK concluded the Trade and Cooperation Agreement (TCA) [25].

The new Trade and Cooperation Agreement sets up a free trade area (FTA) between the EU and the UK. It means that all goods traded between the EU and UK are exempt from tariffs and import quotas. However, there is no specific agreement on non-tariff measures (NTMs). Adversely, EU and British entities face additional regulatory requirements that will make exports of goods more costly and burdensome. In particular there are new rules of origin. EU and UK firms have to determine the origin of their exports to qualify for tariff-free access to the other market. There are limits on what proportion of agri-food products can be assembled from raw materials and ingredients made in third countries in order for them to qualify for tariff-free access (more on rules of origin in preferential trade agreements see e.g. [7, 9, 10, 26–29]). There are also additional testing and certification requirements. But there is no automatic mutual recognition, which means that UK and EU regulatory bodies will not be able to certify products for sale in the EU and the UK, which is potentially a major cost and big obstacle to bilateral trade.

In the agricultural sector, trade between the two sides benefits from the zero-tariff and is not subject to quotas. However, the lack of an equivalence agreement on phytosanitary rules means that exporters face new barriers at the border, including additional border checks. The EU and UK agri-food consignments need to have health certificates and undergo sanitary and phytosanitary controls at member states’ border inspection posts.

The EU and UK will be able to maintain their own sanitary and phytosanitary standards (SPS), although while EU and UK regulations are currently compatible due to EU-driven harmonization, in the longer perspective the SPS standards of the EU and UK may diverge. This process could substantially increase the scope of NTMs between the two parties. Agricultural products from the EU entering the UK will be subject to checks and phytosanitary controls.

The UK was the second largest economy in the EU by GDP. It had very intensive trade relations with the rest of the EU-27, and since leaving the EU has been one of its main non-EU trading partners. The British market is very important to the majority of EU members, including Visegrad countries (V-4). Since the accession of the V-4 countries to the EU, the UK has been gaining in importance as the target export market for all four countries, especially Poland. The Brexit Agreement significantly increased trading costs between the UK and EU member states for all goods, and especially in the agricultural sector. It may have significant negative consequences for agricultural exports of V-4 countries to the UK.

## Literature review

A great deal of previous research has demonstrated that Brexit would cause perturbations in the UK’s agricultural trade relationships with the EU, while there has been a dearth of studies specifically regarding Visegrad countries (V-4). Existing studies include those using both general equilibrium (GE) and partial equilibrium (PE) models, as well as those employing gravity models or just a descriptive approach. No matter what type of methodology was applied, the Brexit scenarios investigated ranged from a WTO-type, hard Brexit relationship in which the UK and EU would trade on Most Favoured Nation (MFN) terms, to an arrangement closer to a free trade agreement or common market (FTA, or “soft” Brexit).

As research to date has shown, the abandoned FTA scenario was expected to result in the least adverse post-Brexit effects in the area of agriculture (as well as in general). Felbermayr et al. [30] used a structural gravity model and estimated an increase in trade frictions under three different scenarios. According to their results, the soft Brexit scenario would have a negligible effect on international trade for EU exports to the UK as well as vice versa. On the other hand, a hard Brexit scenario was expected to bring down agricultural trade by 22.7% in the case of EU-UK exports, while the effects on the UK’s agricultural exports were not found to be statistically significant. The adverse trade diversion effects for the EU were expected to be even higher in another scenario, where the UK would additionally form FTAs with third countries.

Bellora et al. [15], using the MIRAGE GE model, showed that a return to WTO rules would bring a decrease in agri-food trade between the EU and UK of around 62%. In their opinion the relative trade impacts would be heterogeneous across countries. Moreover, a very significant decrease in exports to the UK (around 70%) would be experienced by countries having exports concentrated in sectors with the largest tariff and NTMs increases.

According to the analysis by Lawless and Morgenroth [31], which was also based on a WTO scenario, the largest changes in EU-UK trade would be in food-based sectors. The authors estimated falls by 68% for the dairy, eggs and honey sector, and up to 95% for sugar and confectionary. These results correspond to findings by Davis et al. [32] who developed three alternative trade policy scenarios, including FTA, unilateral trade liberalisation (UTL) and WTO rules combined with the presence or absence of direct payments. They demonstrated that changes would depend on the net trade position and/or world prices and range between relatively modest under an FTA scenario and very significant negative impacts under a UTL scenario. Under the FTA scenario, white meat and dairy sectors would be the only ones facing an improvement in the total trade balance, while under the UTL scenario cattle and sheep, dairy, vegetable oils and fats would be such exceptions.

Ciuriak et al. [33], using a general equilibrium model, also found that meat and dairy products would be those sectors of the UK economy that might improve the trade balance of the UK. Both export and import values of these two product groups would be driven down but a decrease in import values would be much stronger. Those results are in line with van Berkum et al. [34] who found similar trends in the UK’s net trade position using a partial equilibrium model.

Davis et al. [35] and Choi et al. [14] also used partial equilibrium models to examine the trade impacts of Brexit on UK agriculture. According to the former, the introduction of MFN tariffs would reduce both imports from the EU and exports from the UK to the EU, but since the UK is a large net importer in the dairy, beef, pig and poultry sectors, the effect on imports would likely outweigh the effects on exports of these commodities. Choi et al. [14] point to the finding that Brexit would have a much larger impact on relative trade patterns in the UK compared to the EU27. Meat and dairy products in both the UK and EU would appear to be most sensitive to the changes in trade facilitation costs.

There have been several country-specific studies showing the overall mild effects of FTA versus harder-Brexit options. For example, Van Berkum et al. [18] show that under the former scenario Dutch exports to the UK and the rest of the world would largely go unaffected, as the changes in export prices would be relatively small. Rojas-Romagosa [17] in another study for the Netherlands shows a larger negative impact of the WTO option on the sectoral exports compared to the FTA option, in which processed food would be more affected than agricultural products.

Similar trade-related impacts of Brexit were also identified in regard to Danish agri-food exports. Yu’s et al. [19], using general equilibrium simulations, show that agri-food exports to the UK would fall by as much as by 48% under the FTA scenario. For example, in the case of the FTA scenario decreases in exports to the UK would be in the order of 44.3% (processed food) to 56.6% percent (milk and dairy products). The decline in total Danish agri-food exports could be limited thanks to the possibilities of redirecting Danish exports within the EU, and because exports to the UK only account for a fraction of total Danish exports. According to Donnellan and Hanrahan [16], the volume of Irish agri-trade might be relatively unaffected in the case of the FTA option, though the cost of doing business with the UK would increase.

It should be emphasized here that the impact of Brexit on agri-food trade in Visegrad countries (V-4) has been insufficiently analyzed to date. It has only been shown that the WTO option could cause severe consequences [21, 22], including a deterioration in price competitiveness of products imported by the UK, especially in the case of bovine, pork and dairy products. In the light of the above-mentioned lack of quantitative analyses, we attempt to analyze the likely trade implications of Brexit for agricultural exports of V-4 countries using a disaggregated partial equilibrium approach. Our analysis is preceded by a brief description of agri-food trade between V-4 countries and the UK in the period prior to the adoption of the EU-UK Withdrawal Agreement.

## Value and structure of agri-food trade between V-4 countries and the UK

**Fig 1:**
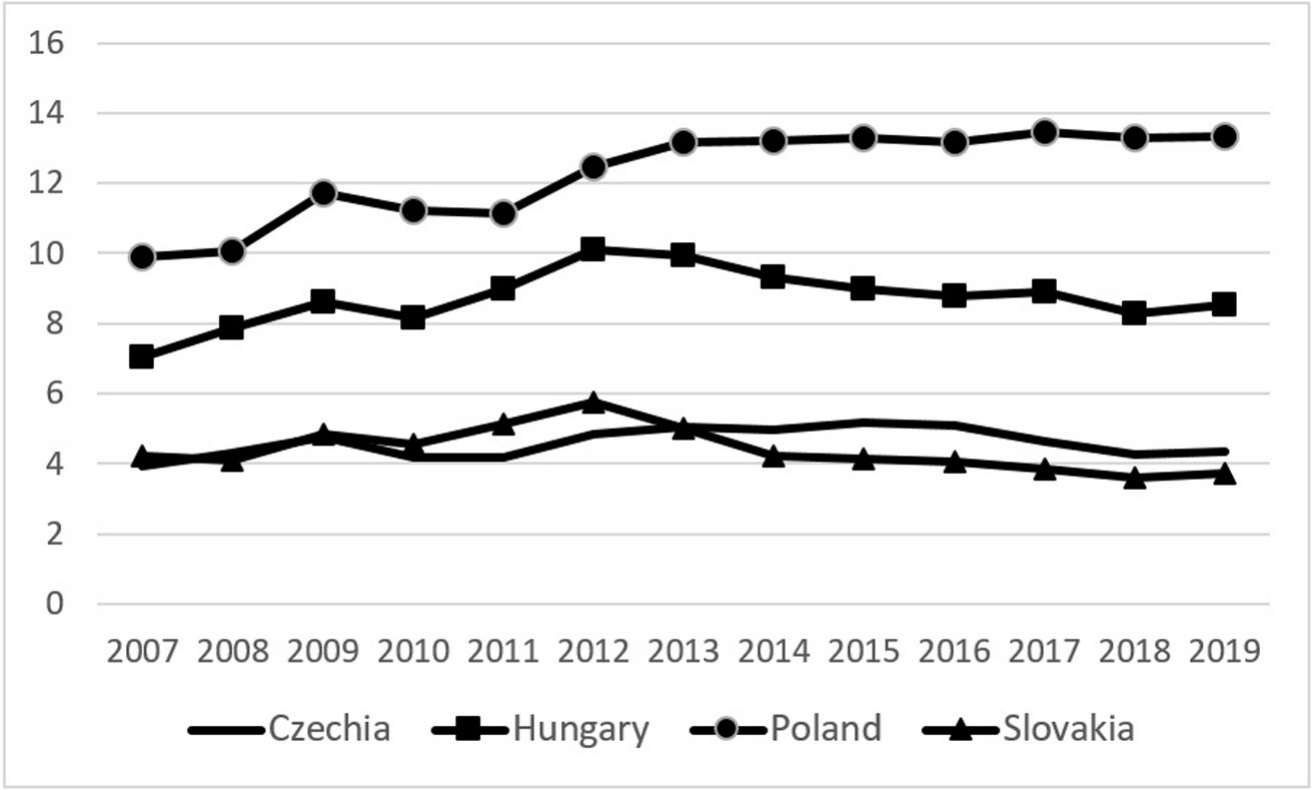
Shares of agri-food products in total commodity exports from V-4 countries in 2007–2019 (%). Source: [36], the authors’ elaboration.

Current studies indicate that Brexit is likely to have significant implications for the agricultural sector and agri-food trade both in the UK and in the countries remaining in the EU, including the group of Visegrad countries (V-4). Agri-food products have always held an important position in the export structure of V-4 countries. Agri-food exports’ share in the total Czech and Slovak commodity exports amounted to around 4–5% in 2007–2019, while in Hungary it reached 7–10%. For Poland, this share was even higher and the value of agri-food exports accounted for 10–13% of the total value of Polish commodity exports (Fig 1). The share of agri-food products in total Polish exports has been rising since the financial crisis of 2008–2009.

Since V-4 countries joined the EU, the UK has been gaining importance as an export destination. In the years 2007–2019 the value of agri-food exports from Poland to the UK almost quadrupled and reached around 3.1 billion USD. In consequence, UK became Poland’s second largest export partner after Germany, and the share of the UK in the structure of the total export of agri-food products of Poland amounted to nearly 9% [36]. It is worth noting that Poland was a net exporter of agri-food products to the UK during the period under consideration. In 2019 agri-food exports from Poland to the UK were almost 17 times higher than imports. The surplus has also been growing over time–by 4.5 times since 2007 (Table 1). A positive and growing trade balance was also observed in the agri-food trade of the other V-4 countries, although the trade surplus values were significantly lower than for Poland, even accounting for differences in country size. The 2019 export values in the other V-4 countries ranged from 40 million USD in Slovakia to 126.5 million USD in Hungary. The relatively high value of agri-food exports from Poland to the UK was chiefly due to the competitive prices, the size of the UK market and consumers’ purchasing power, the relatively small distance to the target market, a similar way of doing business, appreciation of Polish agri-food products in the UK, as well as the high level of consumption of these goods among migrants from Poland.

**Table 1:** Agri-food trade between V-4 countries and the UK in 2007 and 2019 (million USD).

Country	Exports			Imports			Trade balance		
	2007	2019		2007	2019		2007	2019	
	million USD		2007 = 100	million USD		2007 = 100	million USD		2007 = 100
**Czechia**	122.0	114.4	93.8	72.5	58.3	80.4	49.5	56.1	113.4
**Hungary**	120.3	126.5	105.2	55.2	26.1	47.2	65.0	100.4	154.4
**Poland**	828.4	3 072.4	370.9	201.8	182.2	90.3	626.6	2 890.2	461.3
**Slovakia**	39.2	40.6	103.5	28.4	7.3	25.6	10.9	33.4	306.5

Source: [37], the authors’ elaboration.

Agri-food exports from Poland to the UK were not only higher in value, but also much more diversified than from the other V-4 countries. In regard to the 4-digit HS products, the top 5 agri-food products exported from Poland to the UK were: meat and edible offal of poultry; prepared or preserved meat; chocolate and other food preparations containing cocoa; cigarettes; and bread, pastry, cakes, biscuits and other bakers’ wares (Table 2). In 2019 these products were responsible for around 47% of total agri-food exports to the UK. Meat preparation and chocolate along with sugar confectionery, various food preparations and preparations used in animal feeding were mostly exported from Czechia, Hungary and Slovakia. An important item exported from Czechia to the UK was beer (10% of total agri-food exports), while in Slovak exports cheese and curd was of key importance (35% of total agri-food exports). In 2019 the top 5 agri-food products exported from Czechia, Hungary and Slovakia to the UK accounted respectively for 92%, 94% and 99% of their total agri-food exports.

**Table 2:** Top 5 agri-food products exported from V-4 countries to the UK in 2019.

**Czechia**			**Hungary**		
**HS item**	**Million USD**	**Share in total agri-food exports (%)**	**HS item**	**Million USD**	**Share in total agri-food exports (%)**
**1704 –sugar confectionery**	58.2	50.9	**2309 –animal fodder**	50.2	39.7
**2106 –various food preparations**	15.7	13.7	**1602 –meat preparations**	34.2	27.1
**2203—beer**	11.7	10.2	**2106 –various food preparations**	15.6	12.3
**1806—chocolate**	11.1	9.7	**1806—chocolate**	11.3	8.9
**2309 –animal fodder**	8.2	7.1	**1704 –sugar confectionery**	7.6	6.0
**Poland**			**Slovakia**		
**HS item**	**Million USD**	**Share in total agri-food exports (%)**	**HS item**	**Million USD**	**Share in total agri-food exports (%)**
**0207 –poultry meat**	394.4	12.8	**1806—chocolate**	17.1	42.0
**1602 –meat preparations**	350.4	11.4	**0406 –cheese and curd**	14.4	35.4
**1806—chocolate**	293.7	9.6	**1704 –sugar confectionery**	4.4	10.9
**2402—cigarettes**	212.4	6.9	**2106 –various food preparations**	3.9	9.7
**1905 –bakers’ wares**	181.4	5.9	**1602 –meat preparations**	0.3	0.7

Source: [37], the authors’ elaboration.

It is expected that the degree to which Brexit affects trade will be proportional to the increase in non-tariff barriers and trade costs, which are higher for trade in products of animal origin. Due to the relatively high trade share and level of market protection, the biggest drops in exports of agri-food products from all V-4 countries to the UK may be experienced in regard to animal origin products including dairy products, meat preparations or preparations used in animal feeding. It is worth stressing that this observation is in line with the assessment of the impacts of Brexit on Dutch and Danish agri-food trade flows by Yu et al. [19], Bellora et al. [15] and van Berkum et al. [18].

## Methodology

### The analyzed scenario

We analyze a scenario that reflects a conservative outcome of Brexit negotiations. We assumed no changes in the bilateral tariff levels resulting from establishing the free trade area (FTA) covering all products, including agricultural ones. We also assumed no changes in UK tariff levels towards third countries, i.e. the UK maintaining all FTAs already signed. We assumed that the UK applies the same tariffs of the EU Common Customs Tariff (CCT) towards all countries in the world (with the exception of EU members). Moreover, in our simulations the tariff preferences within the Generalized System of Preferences (GSP) towards developing countries are kept unchanged. The same applies to tariffs towards the members of the European Economic Area (EEA) and Switzerland. Of course, in the longer run the UK can sign new agreements liberalizing trade with other countries, and in particular with the US and Australia. In the past, the UK Government frequently complained about the high level of tariff protection of the EU agri-food market. Signing those preferential trade agreements with non-EU countries would probably increase UK imports from non-EU countries and exacerbate Brexit’s adverse effects on the UK’s trade with EU members.

Meeting the requirements of the Brexit Agreement (BA), we assumed that the changes in the level of British trade protection result from the changes in the tariff equivalents of non-tariff measures. Indeed, the BA has no provisions regarding continuation of policy in the field of application of common sanitary and phytosanitary standards (SPS). The BA contains only a general reference to the WTO SPS agreement, which calls for international cooperation and refers to the Codex Alimentarius standards. We therefore assumed that EU and UK SPS standards could diverge. The divergent SPS standards and other technical regulations regarding market access will create additional barriers, i.e. costs that exporters will have to face. We assumed that the level of external non-tariff protection (NTM) in the UK agricultural sector will increase by 25% of the difference in the level of estimated NTM protection in the intra-EU trade and the external NTM tariff equivalents of the EU. This assumption is in line with the idea that while the regulations and standards are going to slightly diverge–due to cultural, economic and geographical distance–this divergence will be limited in the short term, and the UK technical and sanitary requirements will still be closer to EU ones than those employed by third countries.

The NTM tariff equivalents were estimated using the gravity model methodology. We used the GTAP data as a source of bilateral trade data for a panel of two time periods, i.e. 2011 and 2014 (the last available reference years covered by the GTAP 10 Data Base). Data on standard gravity macro variables come from World Development Indicators, while the time-invariant gravity variables (i.e. distances, contiguity, common language, and colonial ties) come from the CEPII geo-dist database. The estimates of reporter-level fixed effects provide an average level of imports of a particular reporter when all other gravity variables are accounted for. Therefore, a difference between country *i* fixed effect and some reference country fixed effect provides, *ceteris paribus*, an approximate percentage deviation in trade between that country and a reference country. We choose the reference country to be the most liberal country in the sample, i.e. having the highest importer-level fixed effect. When we obtain the average fixed effects for all countries, we select the reference country for each sector and compute the average differences between the reporter fixed effects of the EU countries and those of the reference country. Then, using GTAP sectoral Armington elasticity, we recover the $t_{i}^{s}$ –the tariff equivalent of NTMs. Following the methodology employed by Hagemejer et al. [13] we estimate NTM tariff equivalents for the agricultural sectors as defined by the GTAP database. The results of the estimations of NTM tariff equivalents are presented in the “Supporting information” section (S1 Table).

As we simulate the implications of the exclusion of the UK from the Single European Market, we also assume an increase in border costs for exporters. By now the exporters have to meet additional formal requirements, fill additional administrative forms, and TIR trucks are subjected to border control, frequently requiring a couple of hours. These additional formalities entail additional costs, which can be particularly high in the case of animal products. Therefore, we assume that border costs after Brexit increase by 2% on average, and by 5% in the case of animal products. This is in line with the approach by Davis et al. [32]. We combine both changes reflecting the increase in NTM tariff equivalents and increase in border costs in one simulation for sensitive products.

### Specification of the model

We studied the effects of Brexit on agricultural trade using the GSIM partial equilibrium model elaborated by Francois and Hall [38]. The detailed structure of the GSIM model is presented in Jammes and Olarreaga [39]. This partial equilibrium model is grounded on the Armington [40] assumption concerning a constant elasticity of substitution sub-utility function. The representative consumer in an importing country consumes a product being a bundle of different varieties, imported from various countries. Jammes and Olarreaga [39] describe a simpler version of the SMART model and assume a quasi-linear additive utility function that is also additive on a composite numéraire good (n). The aggregate consumer utility function in an importing country is (we follow the notation of Jammes and Olarreaga [39]):

$$U=\sum_{g}u_{g}(m_{g})+n$$ (1)

where *n* is the consumption of the composite numéraire good, *m*_*g*_ is the consumption of imported aggregate good (existing in many varieties from different countries) of good *g*, and *u*_*g*_ is the constant-elasticity of substitution sub-utility of good *g*. The maximization of utility function (1), taking into consideration the budget constraint, gives the Eq (2):

$$m_{g,c}=f(p_{g,c}^{d};p_{g,w}^{d}),\forall g,c$$ (2)

$$n=y-\sum_{c}\sum_{g}p_{g,c}^{d}m_{g,c},$$

where *m*_*g*,*c*_ means the imports of good *g* from country *c*, *p*_*g*,*c*_^*d*^ is the domestic price of imported variety *g* from country *c*, and *p*_*g*,*w*_^*d*^ is the domestic price of good *g* imported from all countries with the exception of *c*, and *y* is the national income. The consumption of the composite and numéraire good absorbs all income effects. In the open economy the domestic price is given by: $p_{g,c}^{d}=p_{g,c}^{w}(1+t_{g,c})$, where the *p*_*g*,*c*_^*w*^ is the world price of good *g* imported from country *c*, and *t*_*g*,*c*_ is the ad valorem tariff imposed on good *g* from country c. Then one can define the trade creation (TC_g,c_) effect expressed in world prices as follows:

$$TC_{g,c}=p_{g,c}^{w}dm_{g,c}=p_{g,c}^{w}\varepsilon_{\text{g},\text{c}}m_{g,c}\frac{dp_{g,c}^{d}}{p_{g,c}^{d}}.$$ (3)

where ε_g,c_ is the price elasticity of import demand and *dm*_*g*,*c*_ is the change in the demand for import of good *g* from country *c*. Using the definition of domestic price ($dp_{g,c}^{d}=p_{g,c}^{w}dt_{g,c}$) and inserting it to (3), and assuming that $p_{g,c}^{w}=1$, we get a formula of TC for calculations.


$$TC_{g,c}=p_{g,c}^{w}dm_{g,c}=p_{g,c}^{w}\varepsilon_{\text{g,c}}m_{g,c}\frac{dt_{g,c}}{\left(1+t_{g,c}\right)}=\varepsilon_{\text{g,c}}m_{g,c}\frac{dt_{g,c}}{\left(1+t_{g,c}\right)}.$$ (4)

If the (non)tariff equivalent change from country *c* (like the UK) is an equivalent of non-preferential tariff increase, then imports of these goods from other countries are going to replace imports from customs union partners (EU), because products from the latter become relatively more expensive. We can also define the trade diversion effect.

Taking into account relative tariff changes, resulting from increases in (non)tariff measures, and recalling the definition of trade diversion *dm*_*g*,*c*_ = − *dm*_*g*,*w*_, we can define the trade diversion as:

$$TD_{g,c}=dm_{g,c}=\frac{m_{g,w}m_{g,c}}{m_{g,c}+m_{g,w}}\frac{dt_{g,c}}{\left(1+t_{g,c}\right)}\sigma_{g,c,w}$$ (5)

where (*σ*_*g*,*c*,*w*_) is the elasticity of substitution, across imports of good *g* from country *c* and all other countries. An additional constraint must be introduced since the trade diversion cannot be larger than the original imports of good *g* from other countries, not *c*.

The simulated changes in the price of a given variety, resulting from changes in tariffs (or non-tariff equivalents), affect the price index and the structure of consumption of different varieties. Thus, by using exogenously given elasticities of export supply, the import demand elasticity and the elasticity of substitution, across imports, it is possible to simulate changes in the trade flows of a given good in many “country specific” varieties. The model considers only the effects of a given policy in the given market and does not account for other economic interactions. This relatively simple partial equilibrium model makes it possible to simulate the effects of changes in tariffs and non-tariff equivalents at a high level of disaggregation.

We applied the GSIM model to analyze the potential trade implications of Brexit for Visegrad countries. We studied the implications of non-tariff increases in British imports originating in EU countries. We analyzed changes in import prices of goods imported from the EU (*own price effect*) and changes in exports from non-EU countries to the UK (*cross price effects*) under the assumption of exogenous world prices. The own price effects and the cross-price effect correspond to trade creation and trade diversion effects, respectively. The increase in British non-tariff measures reduces the imports from the EU countries (negative trade creation) and leads to the substitution of imports from the EU by imports from third countries (negative trade diversion for the EU countries).

We used the standard supply elasticities provided by the GSIM model in the version published on the World Bank’s WITS website. Essentially these elasticities are the update of the elasticities provided in Kee et al. [41]. The elasticity of export equals to infinite (which means setting it to 99). This assures that the exporting country is a price taker in the export market, while elasticities of import demand are different for a given HS6 good and each importing country. On the other hand, we based Armington elasticities of demand on the GTAP database. Finally, the NTM tariff equivalents were based on gravity estimations, presented in an earlier section of this study. The main drawback of this approach is that the NTM tariff equivalents were calculated for broad groups of products within the GTAP classification, while the simulations were performed for more disaggregated 4-digit product groups. The GSIM model simulations were performed for sensitive product groups identified in the next section of the paper and are based on 2020 trade flows and matched to relevant categories of the GTAP classification.

### Identification of the sensitive products

In order to streamline our analysis, we focus our attention on the most sensitive products, the exports of which may be most affected by Brexit. We defined them as being subject to a high level of external Single European Market (SEM) protection (over 30%) and contributing significantly (over 0.5%) to overall exports to the UK from the countries in question.

The trade shares presented in Table 2 are one of three basic ways to look for products that appear to be most sensitive to the changes in trade costs. It is also possible to use market protection level or trade shares along with market protection level to identify sensitive products. When taking into account the level of tariff protection of the EU market, we can see that the highest MFN applied rates (weighted average, incl. AVE) are imposed on dairy products, sugar, meat and edible meat offal, including beef, and manufactured tobacco and tobacco substitutes [42]. However, it should be noted here that in agri-food trade non-tariff measures (NTMs) are an even more serious obstacle to trade growth than tariffs, and should also be included in the investigation. That is why the most comprehensive way for identifying sensitive products is to simultaneously employ trade shares and market protection rates covering both tariffs and non-tariff barriers to trade.

**Fig 2:**
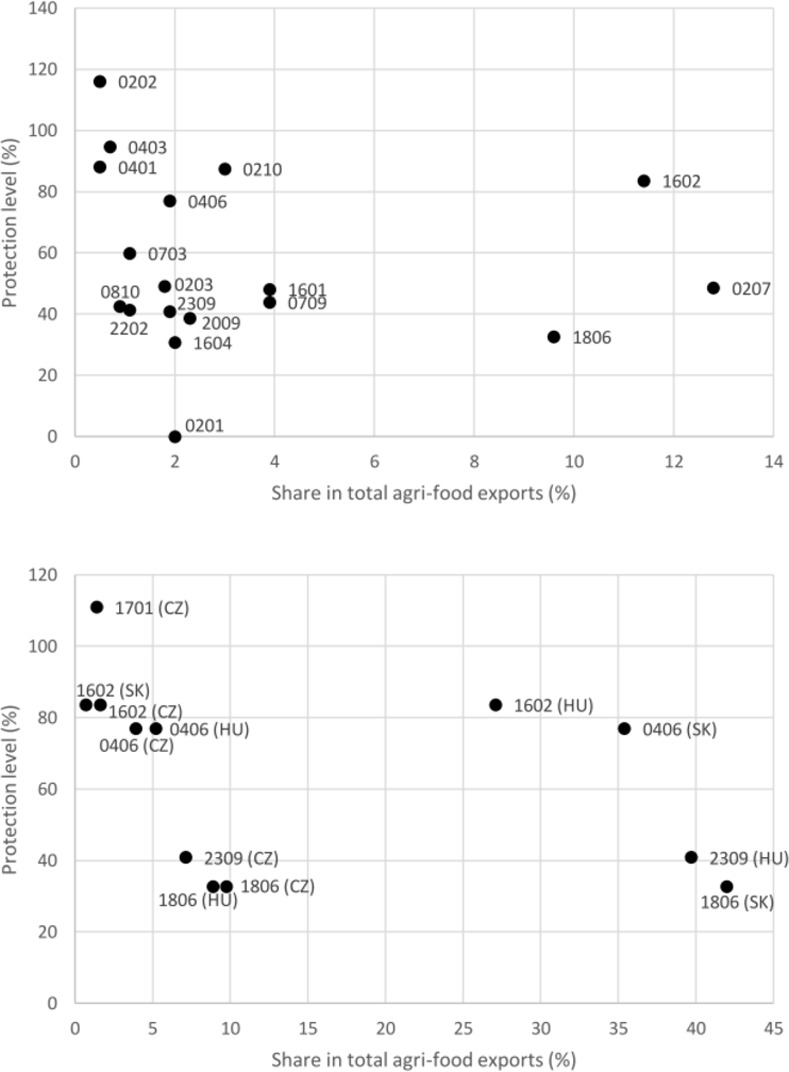
Sensitive agri-food products in exports from V-4 countries to the UK in 2019 (by export shares and market protection rates) for Poland (upper panel) and the other three V-4 countries (lower panel). Source: [13, 36, 42], the authors’ elaboration. HS codes: 0201 –meat of bovine animals; fresh or chilled; 0202 –meat of bovine animals; frozen; 0203 –meat of swine; fresh, chilled or frozen; 0207 –meat and edible offal of poultry; 0210 –meat and edible meat offal; salted, in brine, dried or smoked; 0401 –milk and cream, not concentrated; 0403 –buttermilk, curdled milk and cream, yoghurt, kephir, fermented or acidified milk or cream; 0406 –cheese and curd; 0703 –onions, shallots, garlic, leeks and other alliaceous vegetables; 0709 –vegetables, n.e.s.; 0810 –fresh fruit, n.e.s.; 1601 –sausages and similar products of meat, meat offal or blood; 1602 –prepared or preserved meat, meat offal or blood; 1604 –prepared or preserved fish; 1701 –cane or beet sugar; 1806 –chocolate and other food preparations containing cocoa; 2009 –fruit juices and vegetable juices; 2202 –waters; 2309 –preparations of a kind used in animal feeding.

Taking this approach, we can conclude that the UK market for beef and dairy products features the highest level of protection. In this case, the overall level of market protection ranged from 77% for cheese and curd to over 116% for frozen beef. It was slightly lower for trade in tobacco products, and in the trade of pork, poultry and some fruit and vegetables it ranged from over 42% to 50% (Table 3). Considering both criteria in parallel and assuming over 0.5% share in exports and over 30% overall level of market protection, meat and edible offal, especially beef, poultry, dairy products, certain fruits and vegetables, fish products, chocolate, mineral waters and fruit juices, as well as preparations used in animal feeding, may prove particularly susceptible to the decline in exports from Poland to the UK (Fig 2 upper panel). In 2019 those 18 sensitive tariff lines accounted for over 60% of total agri-food exports from Poland to the UK. The same criteria applied to agri-food exports from the other V-4 countries showed that only a few products, such as cheese and curd, meat preparations, chocolate and preparations used in animal feeding (CZ, HU) and sugar (CZ), should be considered sensitive (Fig 2 bottom panel). These agri-food items were responsible for around 24% (CZ) or 80% (HU, SK) of total agri-food exports to the UK.

**Table 3:** Four-digit tariff groups facing the highest level of tariff and NTMs external protection.

Four-digit tariff group	MFN tariff	NTMs tariff equivalents	Overall level of protection
Meat of bovine animals; frozen	73.80	42.32	116.12
Milk and cream; concentrated	67.76	40.00	107.76
Meat of bovine animals; fresh or chilled	60.43	42.32	102.75
Buttermilk, curdled milk and cream, yoghurt, kephir, fermented or acidified milk or cream	55.04	39.67	94.71
Milk and cream; not concentrated	48.43	39.67	88.10
Meat and edible meat offal; salted, in brine, dried or smoked	61.12	26.40	87.52
Prepared or preserved meat, meat offal or blood	57.29	26.40	83.69
Cheese and curd	37.33	39.67	77.00
Manufactured tobacco and manufactured tobacco substitutes n.e.s.^a^	42.79	30.55	73.34
Onions, shallots, garlic, leeks and other alliaceous vegetables	24.44	35.48	59.92
Cigars, cheroots, cigarillos and cigarettes^a^	28.69	30.55	59.24
Edible offal of bovine animals, swine, sheep, goats, horses, asses, mules or hinnies; fresh, chilled or frozen	13.83	42.32	56.15
Meat of swine; fresh, chilled or frozen	22.81	26.40	49.21
Meat and edible offal of poultry; fresh, chilled or frozen	22.17	26.40	48.57
Sausages and similar products of meat, meat offal or blood	21.77	26.40	48.17
Vegetables; n.e.s. fresh or chilled	8.44	35.48	43.92
Fruit, fresh; n.e.s.	7.05	35.48	42.53

Source: [13, 42], the authors’ elaboration.

Note: a–we excluded these tariff groups from further analysis as we assume that food industry covers manufacture of food products (NACE C10) and beverages (NACE C11).

We can see that the Polish structure of sensitive agricultural exports to the UK is fairly differentiated and covers eighteen 4-digit commodity groups. Many of them are raw plant materials and animal products facing a high level of protection. This situation reflects the large potential of Polish agriculture. On the other hand, the structure of the other three Visegrad countries is much more concentrated. They specialize in one or two 4-digit commodity groups, such as cheese (HS 0406) and chocolate (HS 1806) in the case of Slovakia, or preparations of a kind used in animal feeding (HS 2309) and prepared or preserved meat (HS 1602) in the case of Hungary.

## Results and discussion

Having identified the sensitive products, we performed the simulations for all sensitive products for the four Visegrad countries (V-4). As mentioned in the previous section, the level of post-Brexit border costs adopted in our partial equilibrium simulation was assumed at 2% for plant origin sensitive products and 5% for animal origin sensitive products (S2 and S3 Tables). The results of the short-run simulations for Poland’s exports of agricultural products to the UK are shown in Table 4.

**Table 4:** The simulation of trade flow changes in Poland’s exports of sensitive products to the UK.

Commodity group	4-digit HS code	NTM tariff equivalent	Exports (thousand US$)	Trade creation effect (thousand US$)	Trade diversion effect (thousand US$)	Total trade effect (thousand US$)	Percentage change
**Meat and meat products**	**0201**	42.3	79 373	-11 107	-1 196	-12 304	-15.5
	**0202**	42.3	13 356	-1 498	-320	-1 818	-13.6
	**0203**	26.4	49 468	-4 878	-37	-4 915	-9.9
	**0207**	26.4	377 851	-34 983	-3 344	-38 326	-10.1
	**0210**	26.4	5 219	-866	-326	-1 192	-22.8
**Dairy products**	**0401**	39.7	6 939	-959	0	-959	-13.8
	**0403**	39.7	16 598	-2 280	-2	-2 282	-13.7
	**0406**	39.7	56 036	-6 946	-56	-7 002	-12.5
**Vegetables**	**0703**	35.5	25 164	-7 800	-946	-8 746	-34.8
	**0709**	35.5	108 894	-13 964	-200	-14 164	-13.0
**Fruits**	**0810**	35.5	24 286	-22 641	-65	-22 706	-93.5
**Preparations of meat**	**1601**	26.4	56 368	-8 741	-50	-8 791	-15.6
	**1602**	26.4	1 758	-73	0	-73	-4.2
	**1604**	8.5	53 992	-5 294	-812	-6 106	-11.3
**Cocoa and cocoa preparations**	**1806**	8.5	295 783	-13 498	-1 115	-14 612	-4.9
**Fruit juices**	**2009**	8.5	70 190	-1 741	-1 359	-3 100	-4.4
**Mineral waters**	**2202**	30.6	24 840	-1 576	-193	-1 769	-7.1
**Prepared animal fodder**	**2309**	8.5	45 403	-3 098	-707	-3 806	-8.4
**Total**			1 311 518	-141 943	-10 728	-152 671	-11.6

Source: the authors’ simulations.

The simulation results for Poland show that exports from this country to the UK would decrease as a consequence of negative trade creation (Polish products are becoming more expensive because of higher NTMs and border costs) and negative trade diversion, due to the fact that products imported from third countries are becoming relatively less expensive (the prices of products imported from third countries do not change, but become relatively less expensive in relation to the more expensive products imported from the EU; economic effects of preferential trade reflected in trade creation and trade diversion effects were extensively described by Viner [43]). This leads to the conclusion that changes observed in the volume and structure of trade between the EU and UK result from the diversified scale of difficulties when accessing the market (for more on this see e.g. Baldwin and Wyplosz [44]).

The negative trade creation effect is about thirteen times stronger than the trade diversion effect. This means that there are no simple alternatives to products imported from Poland and other EU members, i.e. the consumer is unable to find substitutes for goods originating in the EU, and as a result reduces overall consumption. According to theses estimations the biggest relative decreases in Polish exports would be observed in the case of fruits, n.e.s. (HS 0810), onions, shallots (HS 0703) and meat and edible meat offal (HS 0210). While the first two items had only about 1% share in the structure of exports from Poland to the UK, meat and edible offal–and likewise certain meat products and dairy products–have much higher shares in the exports structure (Fig 2 upper panel). For these tariff lines, the estimated decrease in exports could range from around 10% to 23%, which in absolute terms could mean a loss of revenue from exports to the UK market of 38 million USD for poultry meat (HS 0207), 12 million USD for fresh or chilled meat of beef (HS 0201), almost 9 million USD for sausages and similar meat products (HS 1601), and 7 million USD for cheese and cottage cheese (HS 0406). The overall drop in Polish exports of sensitive agricultural products could be close to 152.7 million USD, i.e. a decrease of about 11.6%. It should be noted here that stronger impacts of post-Brexit trade policy on meat and dairy exports were also predicted by Bellora et al. [15] for the EU-27, Yu et al. [19] for Denmark and by van Berkum et al. [18] for the Netherlands. The same was found for the UK by Gasiorek et al [45] and Choi et al. [14]. Meat and dairy products along with sugars and sugar confectionary, as well as processed vegetables and fruits, were also identified as the most vulnerable in the bilateral EU-UK post-Brexit relations by Lawless and Morgenroth [31].

The results of the short-run simulations for Czech, Hungarian and Slovak exports of agricultural product to the UK are shown in Table 5. Relative changes in prices of agri-food products exported from these countries to the UK, resulting from increasing trade costs covering both higher NTMs and border costs, would result in negative trade creation and trade diversion effects. Similarly, in Poland’s case the total trade effect would be determined by the trade creation effect rather than by trade diversion. The only exemption to this rule is that of sugars and sugar confectionery exported from Czechia to the UK. This is also one of the most affected products under the Brexit conditions. Moreover, relatively high decreases in exports from the other V-4 countries would be noted in the case of cheese and curd (HS 0406). Chocolate (HS 1806), preparations used in animal feeding (HS 2309) or meat preparations (HS 1602) might be less affected by the changes in trade policy rules.

**Table 5:** The simulation of trade flow changes in the Czech, Hungarian and Slovak exports of sensitive products to the UK.

Commodity group	4-digit HS code	NTM tariff equivalent	Exports (thousand US$)	Trade creation effect (thousand US$)	Trade diversion effect (thousand US$)	Total trade effect (thousand US$)	Percentage change
**Czechia**							
**Dairy products**	**0406**	39.7	3 728	-480	-6	-486	-13.0
**Preparations of meat**	**1602**	26.4	44	-2	0	-2	-4.2
**Sugars and sugar confectionery**	**1701**	32.7	1 973	-129	-222	-350	-17.8
**Cocoa and cocoa preparations**	**1806**	8.5	9 927	-544	-47	-591	-6.0
**Prepared animal fodder**	**2309**	8.5	6 129	-320	-95	-415	-6.8
**Total**			21 801	-1 475	-275	-1 750	-8.0
**Hungary**							
**Dairy products**	**0406**	39.7	1 164	-153	-2	-155	-13.3
**Preparations of meat**	**1602**	26.4	14	-1	0	-1	-4.2
**Cocoa and cocoa preparations**	**1806**	8.5	12 073	-671	-49	-720	-6.0
**Prepared animal fodder**	**2309**	8.5	39 780	-2 455	-618	-3 072	-7.7
**Total**			53 013	-3 280	-669	-3 948	-7.4
**Slovakia**							
**Dairy products**	**0406**	39.7	20 505	-2 806	-49	-2 855	-13.9
**Preparations of meat**	**1602**	26.4	54	-2	0	-2	-4.2
**Cocoa and cocoa preparations**	**1806**	8.5	22 185	-780	-97	-877	-4.0
**Total**			42 744	-3 588	-146	-3 734	-8.7

Source: the authors’ simulations.

Czechia, Hungary and Slovakia trade lessen with the UK than does Poland. Since the values of exports from the former to the UK are much lower, the possible trade effects would be proportionally lower as well. The overall decline in exports of sensitive agricultural products from those three countries would probably reach around 9.5 million US$, i.e. about 16-fold less than from Poland. The reduction in exports of Czechia, Hungary and Slovakia in relative terms would also be much lower and close to 8% compared to 11.6% in the case of Poland. However, due to the high concentration of agri-food exports from those three countries to the UK in a few sensitive product groups, Brexit would be very unfavorable for the exporters and could cause a slowdown in these specific agri-food industries. Such a conclusion is in line with insights by Kordos [46] regarding British-Slovak trade relations.

## Conclusions

In this paper we analyze the implications of Brexit for agri-food exports from four Visegrad countries (V-4). Our scenario is based on the outcome of the negotiations regarding the free trade area (FTA) between the EU and the UK, but with no specific commitments on non-tariff measures to trade (NTMs). We simulate a 25% increase in NTMs, resulting from a possible divergence of regulatory standards and an increase in border costs, differentiated by agricultural sectors. We used a partial equilibrium model together with tariff-equivalents of NTMs estimated using a gravity model.

We identified the 4-digit sensitive agricultural product groups for each V-4 country. These products have a large share in the exports of individual countries (over 0.5%) and face a significant increase in NTMs tariff equivalents and border costs. The pattern of sensitive products is different between individual V-4 countries. In the case of Poland, the export structure is quite diversified and covers 18 sensitive groups, while for the other three countries exports are much more concentrated in narrowly defined product categories. We analyzed trade creation and diversion effects of NTMs and changes in border costs.

The simulations reveal that exports of most sensitive product groups from V-4 countries to the UK could decrease by up to 20%. The overall exports of Polish sensitive products could fall by 152.7 million USD corresponding to -11.6%. Due to much lower values of exports from the other three countries to the UK, the overall decrease in exports, in dollar terms, of sensitive agri-food products from Czechia, Hungary and Slovakia would be about 16 times lower than that in Poland. In relative terms it would be a drop of around 8% in exports of sensitive commodity groups from each country.

These results provide a rough estimate of the scale of the drop in trade in sensitive agricultural products between the V-4 countries and the UK. While the macroeconomic importance of this change is not significant as the agriculture and food sectors account for a limited share of the GDP in each of the economies concerned, for the producers of these sensitive goods the export losses are substantial. However, these results should be interpreted with caution. First of all, the results of our simulations are sensitive to the choice of import demand and imports substitution elasticities, as well as to the estimates of non-tariff barriers. Therefore, the results presented here could be treated as stylized facts rather than actual projections. Secondly, the size of the shock to NTMs is also subject to uncertainty. The actual changes in trade flows will depend a lot in the longer run on whether the UK and EU sanitary and phytosanitary standards diverge, and the extent of regulatory cooperation.

Partial equilibrium simulations allowed us to analyze possible changes in trade flows for disaggregated commodity groups, but they did not demonstrate broader implications for the whole economy. Hence the next step of our research could be an assessment of trade-related impacts of Brexit under general equilibrium conditions, allowing for an identification of directions of possible reallocation of agri-food exports from V-4 countries. Analysis of changes in agricultural output and trade resulting from simultaneous changes in trade policy and agricultural support policy would also shed a light on future relations of V-4 countries with the UK in agri-food sector.

## Supporting information

S1 Table Estimated NTM tariff equivalents. Source: the authors’ estimations using GTAP data.
(DOCX)

S2 Table Border costs for Polish exports of agricultural products to the UK by sensitive product groups. Source: the authors’ elaboration.
(DOCX)

S3 Table Border costs for the Czech, Hungarian and Slovak exports of agricultural products to the UK by sensitive product groups. Source: the authors’ elaboration.
(DOCX)
