# Supplementary material for: How big a drop in agricultural exports to the United Kingdom after Brexit? Simulations for sensitive products of four Visegrad countries
Source: PLoS One. 2022 Sep 20;17(9):e0274462. doi: 10.1371/journal.pone.0274462 (PMC9488795; doi:10.1371/journal.pone.0274462)
Supplement: S1 Table — Source: the authors’ estimations using GTAP data. (DOCX) [file pone.0274462.s001.docx]

**S1 Table.** **Estimated NTM tariff equivalents.**

| **Product name** | **NTM tariff equivalent** | | |
| --- | --- | --- | --- |
|  | **Extra-EU** | **Intra-EU** | **Difference** |
| Paddy rice | 20.9 | 0.0 | 20.9 |
| Wheat | 50.9 | 16.5 | 34.4 |
| Cereal grains n.e.s. | 102.5 | 28.3 | 74.3 |
| Vegetables, fruit, nuts | 35.5 | 0.0 | 35.5 |
| Oil seeds | 37.8 | 0.0 | 37.8 |
| Sugar cane, sugar beet | 0.0 | 0.0 | 0.0 |
| Plant-based fibers | 53.3 | 47.5 | 5.8 |
| Crops n.e.s. | 20.4 | 10.5 | 9.9 |
| Bovine cattle, sheep and goat | 53.2 | 35.8 | 17.4 |
| Animal products n.e.s. | 49.8 | 13.4 | 36.5 |
| Wool, silk-worm cocoons | 0.0 | 0.0 | 0.0 |
| Forestry | 47.8 | 28.2 | 19.7 |
| Fishing | 62.3 | 2.4 | 59.8 |
| Minerals n.e.s. | 100.3 | 61.6 | 38.7 |
| Bovine meat products | 42.5 | 0.2 | 42.3 |
| Meat products n.e.s. | 31.4 | 5.0 | 26.4 |
| Vegetable oils and fats | 32.2 | 18.3 | 13.8 |
| Dairy products | 56.9 | 17.2 | 39.7 |
| Processed rice | 49.1 | 0.0 | 49.1 |
| Sugar | 32.7 | 0.0 | 32.7 |
| Food products n.e.s. | 31.2 | 22.8 | 8.5 |

Source: the authors’ estimations using GTAP data.
