# Supplementary material for: How big a drop in agricultural exports to the United Kingdom after Brexit? Simulations for sensitive products of four Visegrad countries
Source: PLoS One. 2022 Sep 20;17(9):e0274462. doi: 10.1371/journal.pone.0274462 (PMC9488795; doi:10.1371/journal.pone.0274462)
Supplement: S2 Table — Source: the authors’ elaboration. (DOCX) [file pone.0274462.s002.docx]

**S2 Table.** **Border costs for Polish exports of agricultural products to the UK by sensitive product groups**.

| **HS4 Code** | **Commodity** | **Border** **costs (%)** |
| --- | --- | --- |
| **0201** | Meat of bovine animals; fresh or chilled | 5 |
| **0202** | Meat of bovine animals; frozen | 5 |
| **0203** | Meat of swine; fresh, chilled or frozen | 5 |
| **0207** | Meat and edible offal of poultry; of the poultry of heading no. 0105, (i.e. fowls of the species Gallus domesticus), fresh, chilled or frozen | 5 |
| **0210** | Meat and edible meat offal; salted, in brine, dried or smoked; edible flours and meals of meat or meat offal | 5 |
| **0401** | Milk and cream; not concentrated, not containing added sugar or other sweetening matter | 5 |
| **0403** | Buttermilk, curdled milk and cream, yoghurt, kephir, fermented or acidified milk or cream, whether or not concentrated, containing added sugar, sweetening matter, flavoured or added fruit or cocoa | 5 |
| **0406** | Cheese and curd | 5 |
| **0703** | Onions, shallots, garlic, leeks and other alliaceous vegetables; fresh or chilled | 2 |
| **0709** | Vegetables; n.e.s. in chapter 07, fresh or chilled | 2 |
| **0810** | Fruit, fresh; n.e.s. in chapter 08 | 2 |
| **1601** | Sausages and similar products of meat, meat offal or blood; food preparations based on these products | 5 |
| **1602** | Prepared or preserved meat, meat offal or blood | 5 |
| **1604** | Prepared or preserved fish; caviar and caviar substitutes prepared from fish eggs | 5 |
| **1806** | Chocolate and other food preparations containing cocoa | 2 |
| **2009** | Fruit juices (including grape must) and vegetable juices, unfermented, not containing added spirit; whether or not containing added sugar or other sweetening matter | 2 |
| **2202** | Waters, including mineral and aerated waters, containing added sugar or sweetening matter, flavoured; other non-alcoholic beverages, not including fruit or vegetable juices of heading no. 2009 | 2 |
| **2309** | Preparations of a kind used in animal feeding | 2 |
| **2402** | Cigars, cheroots, cigarillos and cigarettes; of tobacco or of tobacco substitutes | 2 |
| **2403** | Manufactured tobacco and manufactured tobacco substitutes n.e.c; homogenised or reconstituted tobacco; tobacco extracts and essences | 2 |

Source: the authors’ elaboration.
