# Supplementary material for: How big a drop in agricultural exports to the United Kingdom after Brexit? Simulations for sensitive products of four Visegrad countries
Source: PLoS One. 2022 Sep 20;17(9):e0274462. doi: 10.1371/journal.pone.0274462 (PMC9488795; doi:10.1371/journal.pone.0274462)
Supplement: S3 Table — Source: the authors’ elaboration. (DOCX) [file pone.0274462.s003.docx]

**S3 Table.** **Border costs for the Czech, Hungarian and Slovak exports of agricultural products to the UK by sensitive product groups.**

| **HS4 Code** | **Commodity** | **Border**  **costs (%)** |
| --- | --- | --- |
|  |  |  |
| **0406** | Cheese and curd | 5 |
| **1602** | Prepared or preserved meat, meat offal or blood | 5 |
| **1701** | Cane or beet sugar and chemically pure sucrose, in solid form | 2 |
| **1704** | Sugar confectionery (including white chocolate), not containing cocoa | 2 |
| **1806** | Chocolate and other food preparations containing cocoa | 2 |
| **2106** | Food preparations not elsewhere specified or included | 2 |
| **2203** | Beer made from malt | 2 |
| **2309** | Preparations of a kind used in animal feeding | 2 |

Source: the authors’ elaboration.
